# The Effect of Storage and Pasteurization (Thermal and High-Pressure) Conditions on the Stability of Phycocyanobilin and Phycobiliproteins

**DOI:** 10.3390/antiox12030568

**Published:** 2023-02-24

**Authors:** Hani Shkolnikov Lozober, Zoya Okun, Galit Parvari, Avi Shpigelman

**Affiliations:** 1 Faculty of Biotechnology and Food Engineering, Technion, Israel Institute of Technology, Haifa 3200003, Israel; 2 Faculty of Chemistry, Technion, Israel Institute of Technology, Haifa 3200003, Israel

**Keywords:** phycocyanobilin, phycobiliproteins, stability, pasteurization, high-pressure processing, heat treatment

## Abstract

The utilization of natural blue pigments in foods is difficult as they are usually unstable during processing and the commonly applied pH. The current study focuses on natural blue pigment, possessing antioxidant properties, found in *Arthrospira platensis* (spirulina), and phycobiliproteins (PBP). These pigments are a complex of conjugated protein and non-protein components, known as phycocyanobilin. PBP has low stability during pasteurization (high-pressure or heat treatments), resulting in protein denaturation and color deterioration that limits the application. The phycocyanobilin pigment might also be liable to oxidation during pasteurization and storage, resulting in color deterioration. Yet, the instability of the pigment phycocyanobilin during the pasteurization process and storage conditions was never studied before, limiting the comprehensive understanding of the reasons for PBP instability. In this study, the stability of phycocyanobilin under high-pressure and high-temperature conditions was compared to the stability of phycobiliproteins. We revealed that phycobiliproteins have a higher color deterioration rate at 70–80 °C than at high-pressure (300–600 MPa) whereas phycocyanobilin remained stable during high-pressure and heat processing. During storage at pH 7, phycocyanobilin was oxidized, and the oxidation rate increased with increasing pH, while at lower pH phycocyanobilin had low solubility and resulted in aggregation.

## 1. Introduction

In recent years, the demand for natural pigments has increased due to the health concerns associated with synthetic colorants. Natural red, yellow, and orange pigments such as carotenoids and anthocyanins can be extracted and used in the food industry. Natural blue pigments such as anthocyanins and phycocyanins are abundant in nature; however, their application in foods is utterly difficult due to the instability during processing and the commonly applied pH [1]. Phycocyanins are members of the phycobiliprotein family composed of natural blue pigment and protein complexes commonly found in cyanobacteria. Recently, the FDA approved phycocyanin extract from *Arthrospira platensis* (spirulina) for candy and chewing gum coloring. However, their application is limited due to their high instability at elevated temperatures [1,2]. Phycocyanins were suggested to promote various health benefits such as antioxidant, antiviral, and anti-inflammatory activities [3,4]. Lately, the antiviral properties of phycocyanins have been gaining increased attention due to their reported ability to prevent COVID-19 replication [5]. 

**Figure 1:**
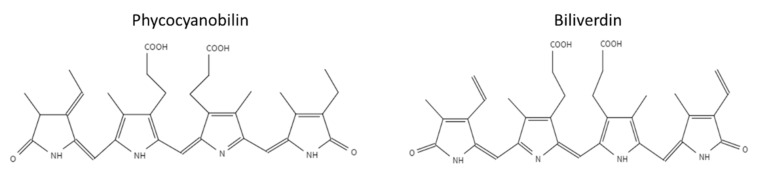
Chemical structures of phycocyanobilin and biliverdin.

Phycobiliproteins (PBP) are light-harvesting proteins covalently bound to phycobilin pigment. Phycobilins are open-chain tetrapyrrole pigments with a structure similar to biliverdin [6,7,8]. Phycobilins biosynthesis is derived from heme metabolism. Heme splits into biliverdin by heme oxygenase (HO), and then biliverdin can be further reduced to phycobilin [6]. Phycobiliproteins are classified into four subclasses according to their light absorption and the Phycobilin type: pink–purple phycoerythrins, blue phycocyanins, orange phycoerythrocyanins, and blue–green allophycocyanins [8]. This study focuses on the stability of PBP from the cyanobacteria *Arthrospira platensis* (spirulina), which is known for its high content of PBP [9]. The two main phycobiliproteins in spirulina are C-phycocyanin (C-PC) and allophycocyanin (APC). C-PC is more abundant in spirulina with an approximate ratio of 10:1 (C-PC: APC) [10,11]. The phycobiliproteins in spirulina contain two subunits α and β, each covalently linked to the pigment phycocyanobilin (PCB). The unique system of conjugated double bonds makes phycocyanobilin an excellent antioxidant [12]. The chemical structures of the tetrapyrrole pigment biliverdin and phycocyanobilin are presented in Figure 1 [13]. The hydrogen atoms of the phycocyanobilin allylic group and amine group (located in the pyrrole rings) can easily be transferred to radicals. Thus, phycocyanobilin might be easily oxidized [11,12,14,15]. However, previous studies have not focused on the degradation of isolated phycocyanobilin, possibly due to the challenging isolation process ascribed to the low concentration of PCB in PBP. Weesepoel et al., 2015 studied light-accelerated biliverdin autoxidation as a free tetrapyrrole pigment model with a structure similar to PCB. The researchers reported that this pigment could undergo autoxidation with various oxidation products, hypothesizing that such oxidation may also occur when the pigment is conjugated to a protein [14]. PBP from spirulina was reported to be highly unstable under elevated temperatures; heating PBP to temperatures higher than 45 °C resulted in its fast degradation [16,17,18,19,20]. The PBP stability is also affected by the pH level, with the highest stability around pH 5–6, while an increase in the pH results in decreased stability [16,17,19]. Near the protein isoelectric point (pH 3–4) the protein is highly unstable and precipitates at room temperature [16].

High-pressure processing (HPP) is a non-thermal treatment commonly used as an alternative to thermal pasteurization. For typical HPP pasteurization, 300–600 MPa is usually applied for less than 5 min, the process can be carried out at room temperature or refrigerated conditions for enhanced quality retention [21,22]. Compared to thermal pasteurization, the main advantage of HPP is that the taste, texture, color, and nutritional value are usually minimally affected by the pressure within the standard pasteurization times [23,24]. Although mostly HPP is not reported to cause the degradation of small molecules such as pigments, some previous studies reported that HPP induced the degradation of anthocyanins and carotenoids [25,26,27]. Usually, high pressure does not affect the protein’s primary structure, while depending on the pressure level, the secondary, tertiary, and quaternary structures may be affected. Typically, at pressure levels of 100–200, MPa proteins may undergo reversible denaturation, while at pressure levels above 300 MPa irreversible denaturation was reported to occur, followed by protein aggregation [28]. The stability of PBP under high pressure has been investigated, and it was detected that HPP causes PBP aggregation and dissociation to subunits [29,30]. Zhang et al. 2021 investigated the stability of C-phycocyanin at various pH levels after HPP treatment at 450 and 600 MPa for 3.5 min. The researchers observed that at pH 5, 25% color deterioration occurred after HPP treatment (at 450 and 600 MPa), while at pH 7, only 7–14% color deterioration was observed. At pH 3 C-phycocyanin remained stable right after HPP treatment; however, extensive color deterioration was detected after one day of storage [31], probably as a result of C-PC limited solubility in this pH [16]. Similar results were detected by Faieta et al., 2021 who investigated the degradation of PBP at aqueous dispersions at 600 MPa for 5 min. The researchers found that high-pressure treatment decreased the content of C-PC and APC by 19% and 35%, respectively. In addition, they reported that sugars have a baroprotective effect (protection from pressure-derived effects) against PBP degradation [30]. 

Recently, the antioxidant activity of PBP from spirulina has gained extensive attention, and many potential health benefits have been discussed [32]. However, it is important to notice that different processes such as extraction, heat, and high-pressure treatments might extensively change the antioxidant properties of PBP [30,32,33] in addition to the described effect on the color properties. Sharma et al. 2021 studied the DPPH radical scavenging of PBP and reported that at high temperatures above 55 °C the ability of PBP to scavenge DPPH radicals decreases [33]. It can be suggested that this is a result of protein denaturation. Another study by Faieta et al., 2021 reported that HPP treatment at 600 MPa for 5 min decreased the trolox equivalent antioxidant capacity (TEAC) of PBP from spirulina by 20% [30].

In this study, PCB was extracted and its stability under high-pressure and temperature conditions was explored and compared to PBP degradation kinetics (at elevated pressure or temperature) to reveal if the degradation of the pigment itself contributes to the color loss. Finally, the stability of extracted PCB at different storage pH levels was also studied. Understanding the mechanisms and kinetics of pigment stability is essential for better utilization of phycocyanins as food pigments. 

## 2. Materials and Methods

### 2.1. Materials

Grounded powder of *Arthrospira platensis* (lot number D1246) was purchased from Abundance (Kfar Rupin, Israel). Biliverdin (CAS registry number 856699-18-8) purity >98% was purchased from Cayman chemical company (Ann Arbor, MI, USA). Sodium phosphate dibasic, sodium phosphate monobasic, sodium citrate dihydrate, and Tris (hydroxymethyl) aminomethane were purchased from Spectrum Chemical (New Brunswick, Canada). Hydrochloric acid, sodium hydroxide, citric acid, methanol absolute CP, ethanol absolute AR, and dichloromethane CP were purchased from Bio-lab (Jerusalem, Israel). Ammonium persulfate was purchased from Sigma Aldrich (St. Louis, MO, USA). HPLC grade acetonitrile was purchased from J.T Baker (Gilwice, Poland). HPLC grade water was purchased from Marcon (Giliwice, Poland). 

### 2.2. Extraction of Arthrospira Platensis (Spirulina) Protein Concentrate (SPC)

The protein concentrate was extracted according to our previously published method [34]. Briefly, spirulina powder was dissolved in distilled water (DW) followed by pH adjustment to 10 by NaOH (0.5 M). Next, the solution was centrifugated (18,500× *g*, 30 min, 25 °C), and the supernatant was collected, the same process was repeated with the pellet. Then, both supernatants were combined, the pH was adjusted to 3 with 0.5 M HCl and the proteins were collected by centrifugation in the same conditions reported above. Finally, the pH was neutralized with NaOH (0.5 M) and freeze-dried.

### 2.3. Preparation of Phycobiliproteins (PBP) Extract 

PBP was extracted from SPC based on our previously published method [35]. Ammonium sulfate (25% (*w*/*v*)) was dissolved in 10% (*w*/*v*) SPC solution in DW and centrifuged for 30 min (25 °C, 10,000× *g*, 25 °C). The precipitate was dissolved again in DW, and 50% (*w*/*v*) ammonium sulfate was added to the solution. The solution was centrifuged again in the same conditions and the precipitate was dissolved in DW, dialyzed to remove ammonium sulfate residues, and freeze-dried. The purity ratio of the PBP was quantified spectrally using synergy H1 microplate reader (Biotek, Winooski, VT, USA) based on a previously published method by Patil et al. (2006) [36]. The protein content was analyzed with CHNS elemental analyzer (Flash 2000, Thermo-scientific, Milan, Italy) based on previously described parameters Chirug et al. (2018) [37] using 6.25 as the nitrogen to protein conversion factor [38].

### 2.4. Preparation of Phycocyanobilin (PCB) Extract 

The cleavage of PCB was based on a previously published method [39] with a few modifications. A 1 (g) amount of SPC was refluxed in 100 mL absolute ethanol for 8 h, followed by filtration with Whatman filter paper number 1 (GE Healthcare, Casoria, Italy). The solvent was then evaporated, and the extract was dissolved in 5 mL ethanol. Silica gel (silica gel 60 (0.063–0.20 mm), Merck, Darmstadt, Germany) was mixed with dichloromethane and loaded in a glass column 3 × 23 (cm). The extract was loaded on the column and eluted with dichloromethane, gradually methanol was added to dichloromethane until the concentration of methanol was 30% (*v*/*v*), in this stage blue fraction was observed visually and collected. The blue eluate fraction was evaporated and analyzed by LC-MS (the detailed procedure is presented in Section 2.7). 

### 2.5. Buffer and Stock Solution Preparation

Baroresistant (pressure stable) buffer was prepared based on the method of Shkolnikov et al. (2020), for the preparation of 100 mL baroresistant buffer: 89.38 mL Tris buffer 100 mM, pH 7 was mixed with 10.62 mL phosphate buffer 100 mM, pH 7 [40,41]. The baroresistant buffer allowed us to avoid pH changes at elevated pressure that interfered with our focus on the impact of pressure only. Phosphate–citrate buffer 100 mM pH 3–7 was prepared using dibasic sodium phosphate and citric acid [42]. Tris buffer 100 mM pH 8 was prepared using tris(hydroxymethyl) aminomethane and hydrochloric acid.

To work at minimal concentrations of methanol in the samples, a stock solution of PCB extract (3% (*w*/*v*) and biliverdin (0.5% (*w*/*v*) were dissolved in methanol, and the solutions were kept at −20 °C until use.

### 2.6. LC-MS Method Development and Analysis of PCB Extract and Biliverdin

Agilent 1260 liquid chromatography system with MS 6120 detector equipped with Gemini column 120 EC-C18 4.6 × 250 (mm) 5-μm was employed for analysis. The autosampler was set to 25 °C, while the column temperature was 30 °C. Before the analysis, the samples were filtered by a 0.45 μm PVDF syringe filter (Merck Millipore Ltd., Carrigtwokill, Ireland). Mobile phases included: an aqueous solution of 1% formic acid (A) and 44% H_2_O, 1% formic acid, and 55% acetonitrile (B). The injection volume was 30 μL and the quantification was carried out at 270 nm and 620 nm. For PCB extract analysis, the following gradients were used after optimization: 75–30% A for 0–2 (min), 30% A for 2–15 (min), 30–50% A for 15–20 (min), 50–75% A for 20–22 (min) and 75% A for 22–25 (min). Flow rate of 1 (mL/min) was employed for the entire procedure. For biliverdin, the following gradients were used: 100% A for 0–1 (min) at flow rate of 1 (mL/min), 100–0% A for 1–5 (min) at flow rate of 1 (mL/min), 0% A 5–5.1 (min) at flow rate of 1–0.8 (mL/min), 0% A 5.1–10 (min) at flow rate of 0.8 (mL/min), 100% A 10–13 (min) at flow rate of 0.8–1 (mL/min) and 100% A 13–15 (min) at flow rate of 1 (mL/min). 

### 2.7. Spectral Stability of PBP Extract, PCB Extract, and Biliverdin after High Pressure

High hydrostatic pressure S-FL-850-09-W system (Stansted Fluid Power Ltd., Harlow, Essex, UK) was used to study the stability of PBP extract, PCB extract, and biliverdin under high pressure. The system’s temperature (25 °C) was controlled using FP51-SL Ultra-Low Refrigerated-Heating Circulator (JULABO GmbH, Seelbach, Germany). PCB extract and biliverdin stock solutions were diluted with baroresistant buffer to 0.02 and 0.002% (*w*/*v*), respectively. PBP extract 0.05% (*w*/*v*) in baroresistant buffer was prepared. Before the measurement, the PBP extract solution was centrifuged for 5 min at 5000× *g* to avoid interference of residual insoluble matter in the extract. Centrifugal tubes of 400 µL were filled with the pigment solutions without leaving headspace and covered with parafilm^®^ to avoid leakage during HPP. Pressure levels of 300, 400, 500, and 600 MPa were applied for 20 min while control tubes with the same solutions were kept at atmospheric pressure in a circulating water bath for 20 min. Immediately after the HPP, 200 µL of the samples were transferred to a quartz microplate, and the UV/Vis absorbance spectrum (270–800 nm) was measured with a synergy H1 microplate reader (Biotek, Winooski, VT, USA). 

### 2.8. Spectral Stability of PBP Extract, PCB Extract, and Biliverdin under High-Temperature Conditions

To study the temperature effect on the stability of PBP extract, PCB extract, and biliverdin, the pigments were heated in a water bath. An amount of 800 µL of pigment solution in baroresistant buffer (prepared in the same concentrations as described in Section 2.7) was filled in a centrifugal tube and heated for 30 s (after reaching 70 and 80 °C) and 30 min (at 60 °C). Immediately after heating, the solutions were frozen in liquid nitrogen to stop the reaction. Afterward, the solutions were defrosted and immediately monitored with a microplate reader as described in Section 2.7. 

### 2.9. Color Deterioration Kinetics of PBP Extract under High Temperature and Pressure

The absorbance spectrum of freshly prepared PBP extract 0.05% (*w*/*v*) in baroresistant buffer solution (prepared by the same method as described in Section 2.7) at constant pressure (300–600 MPa, 25 °C during 20 (min)), and constant temperature (60, 70, and 80 °C, atmospheric pressure during 460, 80 and 40 (s), respectively), were recorded in situ by the use of laboratory-scale high-pressure sterilization unit model 765.0600, SITEC High-Pressure Technology. The temperature was kept constant using a water-circulating chiller (Hakke DC30/K10). The system includes a high-pressure optical cell (Type 740.2330 from SITEC Sieber Engineering AG, Switzerland). The optical cell with a pathway of 10 mm was connected to a UV–Vis spectrometer (Evolution 260, Thermo Scientific, Waltham, MA, USA) by optic fiber cable [41]. 

### 2.10. The Stability of Biliverdin and PCB Extract at Room Temperature

The stability of biliverdin (0.002% (*w*/*v*)) and PCB extract (0.02% (*w*/*v*)) in citrate/phosphate buffer pH 7 100 mM and Tris buffer pH 8 100 mM was monitored. Glass vials were kept in LC-MS autosampler set to 25 °C. The samples were injected for LC-MS analysis every 0.5–12 h to obtain at least 6 time points. The analysis was carried out by the above method (Section 2.6). 

### 2.11. PCB Extract Aggregation at Various pH Levels

The aggregation of 0.02% PCB extract and 0.002% biliverdin at pH 3, 5, 7 (citrate/phosphate buffer 100 mM), and pH 8 (Tris buffer 100 mM) were studied using Primo star microscope (Zeiss, Oberkochen, Germany). A 40× primo plan ACHROMAT objective and 10 XWF eyepieces were used to gain 400 total magnification. For detection of solubility decrease, the samples were centrifuged for 2 (min) at 2000 g using MLX-110 mini centrifuge (Crystal, Addison, Arizona), and the UV/Vis absorbance spectrum was measured with Ultospec 2000 (Pharmacia Biotech, Chicago, IL, USA). 

### 2.12. Verification of PCB Extract Oxidation Mechanism at pH 7 and 8

To verify that PCB degradation is spontaneous oxidation due to exposure to the air oxygen, nitrogen gas was purged into citrate/phosphate buffer 100 mM pH 7 and Tris buffer 100 mM pH 8 for 15 min. Afterward, PCB extract stock solution was added to the buffer to reach a final concentration of 0.02%. Then, nitrogen was purged again for 3 min. The solution was closed immediately in a glass vial to ensure an inert atmosphere and kept dark at room temperature until analysis. As a control, the same solution was examined without nitrogen purging. The absorbance at pH 7 and 8 after 2 (h) and 29 (h), respectively, was monitored by a microplate reader by the method described in Section 2.7. PCB extract solution at time 0 h was also analyzed as a control.

### 2.13. Statistical Analysis

All processing was repeated at least twice using freshly prepared samples. The number of full repetitions is presented for relevant figures/tables. One-way ANOVA with post hoc Tukey (honestly significant difference) test was performed to determine statistically significant differences.

## 3. Results

### 3.1. Characterization of PBP and PCB Extracts

Phycobiliprotein (PBP) extract was obtained from spirulina protein concentrate by ammonium sulfate precipitation. The protein content of the extract was 80.5 ± 0.2 (% *w*/*w*). Purity was determined as the absorbance ratio, which defines the relationship between the presence of the desired compound and other proteins present [43]. The absorbance purity ratio for CPC was calculated as A620 nm/A280 nm revealing the value of 1.8 ± 0.09, while the purity ratio for APC was 0.91 ± 0.04 (A652/A280). 

**Figure 2:**
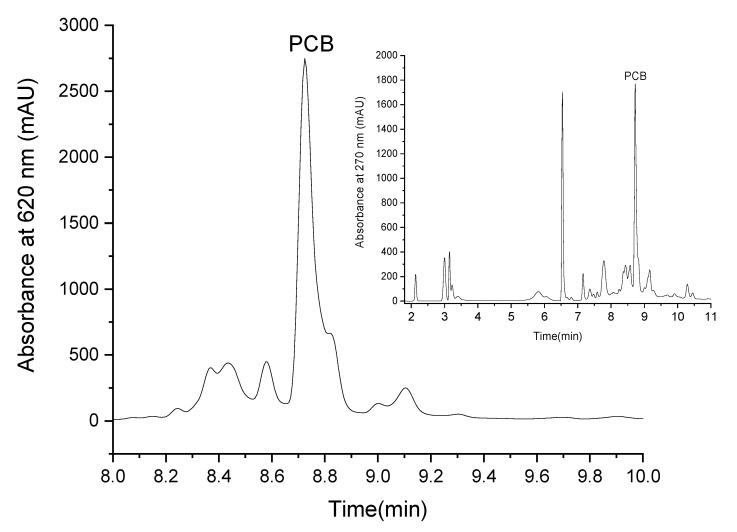
LC elution profile of PCB extract at 620 nm (the inset presents the elution profile of PCB extract at 270 nm).

PCB extract was isolated by ethanol reflux extraction. LC-MS analysis revealed that the PCB purity at 620 nm was 83 ± 4% while at 270 nm it presented 30.8 ± 0.3% from the extract. The blue pigment can be defined as a molecule that absorbs red light (in the region of 600 nm) [1]. The high purity of our extract at 620 nm indicates that the extract’s blue color results mainly from PCB, whereas the lower purity at 270 indicates that the extract also contains other materials, but they mainly do not absorb at 620 nm and do not contribute to the extract’s blue color. Figure 2 presents the chromatogram of PCB extract at 620 nm (the chromatogram of PCB extract at 270 nm is presented in Figure 2 as an inset) and reveals that the prominent peak at this wavelength is PCB (this was also verified by MS-ESI detector). The PCB peak split is possibly due to the formation of two isomers [39] of a similar polarity.

### 3.2. The Stability of PBP Extract and PCB Extract under Various Pasteurization Conditions

Usually, high-pressure pasteurization possesses no adverse effect on the stability of small molecules such as the explored pigment. Nevertheless, in some cases, it was reported that pressure pasteurization of small molecules such as carotenoids and polyphenols results in their degradation [25,26,27]. Previous studies reported that due to denaturation and aggregation, HPP and heat treatment resulted in PBP color deterioration [17,30,31]. Currently, no information exists on the effect of pressure and high temperature on the stability of the pigment PCB itself. Thus, as PCB might be sensitive to oxidation, we hypothesized that, similar to other natural pigments it might degrade during thermal treatment and possibly even due to high pressure. Such degradation might indicate that the pigment also degrades while bound to the protein, potentially contributing to color loss during processing. In addition, because the extracted pigment is not entirely pure, the stability of biliverdin was studied as a model. Biliverdin was previously suggested to be a high-purity free tetrapyrrole pigment with a structure similar to PCB [14]. It should be mentioned, that due to the structural differences between PCB and biliverdin (Figure 1), the degradation rate under the explored storage and pasteurization conditions might differ. The heat and high-pressure effect on PBP extract, biliverdin, and PCB extract were studied at pH 7. In this pH, all pigments have relatively high solubility, thus enabling spectrophotometric monitoring and analysis. 

**Figure 3:**
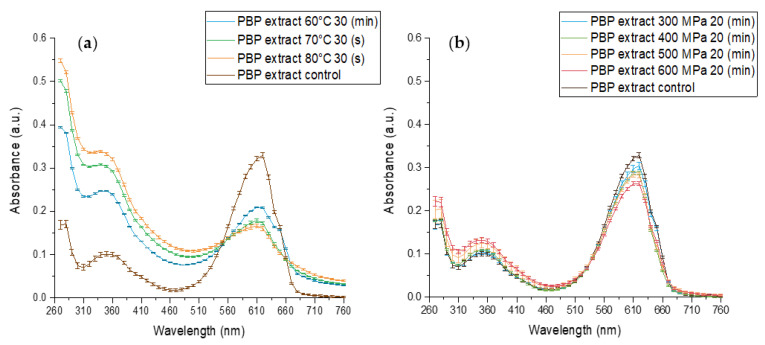
The effect of different high-pressure and temperature treatments on the stability of PBP extract 0.05 (% *w*/*v*) in baroresistant buffer pH 7 100 mM, control treatment at atmospheric pressure 25 °C (**a**) The effect of high-temperature (**b**) the effect of high-pressure. (*n* = 2, error bars represent S.E.).

The effect of standard heat pasteurization conditions at 70, 80 °C for 30 (s) and 60 °C for 30 min on PBP extract stability at pH 7 is presented in Figure 3a and reveals an extensive change in absorbance. The absorbance maxima at 620 nm decreased while the maximum absorbance at 360 and 280 nm increased. The extent of the alterations increased with increasing temperature, indicating a temperature-dependent decrease in blue color. At the same time, an increase at 280 nm is likely indicative of protein structural changes and possible protein denaturation. In its native form, when phycocyanobilin is bonded to phycobiliproteins, the pigment is forced into energetically unfavorable starched conformation. In this conformation, an extended double-bound system is formed by the protein and the pigment, and C-phycocyanin (the main PBP) has a maximum absorbance of 620 nm. However, when the protein denatures the pigment may rearrange to energetically favored cyclic conformation. In this conformation, phycocyanobilin has a maximum absorbance of 360 nm. Thus, with the simultaneous increase at the 360 nm peak and decrease at the 620 nm peak, we have observed protein denaturation resulting in pigment conformational change from stretched to cyclic. Those results align with the known impact of processing on the UV absorbance of phycocyanins and proteins [44,45,46]. 

The effect of high-pressure treatments on PBP extract stability is presented in Figure 3b. Similar to the heat effect on the PBP extract absorption spectrum, the pressure treatment resulted in a decrease at 620 nm absorbance and an increase at 360 and 280 nm absorbance. Thus, HPP resulted in PBP extract color deterioration, pigment conformational change, and protein denaturation. These effects were more pronounced as the pressure increased. A similar HPP direction of effect on PBP extract was observed previously. Zhang et al., 2021 revealed that HPP treatment for 3.5 (min) at 450 and 600 MPa resulted in color deterioration.

However, when comparing HPP and thermal treatments, we observed that after 20 min at 500 and 600 MPa, which is a much more intensive treatment than commercially used for HPP to reach pasteurization level treatments, spectrally, the negative impact seems much smaller than for 70 °C for 30 (s), the intensity of mild pasteurization. On the other hand, the difference in absorbance spectra and the presence of isosbestic points in the case of heat treatment point toward a hypothesis that the mechanism of protein denaturation during HPP might be substantially different from the denaturation during heat treatment. Such an observation expands and supports previous reports of suggested mechanism differences [47,48]. In addition, researchers revealed irreversible protein denaturation and aggregation during HPP and heat treatment [30,31]. 

In addition to the negative impact of processing on color, PBP deterioration during high-pressure and heat treatments was reported to be correlated with a loss of antioxidative activity, emphasizing the importance of minimizing PBP deterioration. Some suggest adding stabilizers such as sugars to minimize this effect [30,49,50]

The effect of heat and pressure treatment on biliverdin and PCB extract stability is presented in Supplementary Materials Figures S1 and S2, respectively. At all treatments, the pigments, PCB, and biliverdin remained stable during processing; thus, excluding pigment degradation as a contributing factor to the processing-induced color loss. The high stability of PCB during heat and pressure treatments might imply its potential as a food pigment, from the processing point of view. However, to further implement PCB as a food pigment, the economic and environmental effects of spirulina utilization as a potential source for commercial applications is needed. Furthermore, the utilization of circular food supply chain methodology and waste stream collection for recycling and generation of new products and valuable components, resulting in a sustainable production system of such pigments, should also be explored and addressed in future studies [51,52].

### 3.3. The Kinetics of PBP Extract Color Deterioration at Different Pasteurization Treatments 

In the previous section, we have revealed that heat and high-pressure treatment of PBP extract results in an absorbance decrease at 620 nm. Such a decrease is likely a result of protein denaturation and aggregation. While previous works have reported similar effects on PBP extract stability, these works focused on the HPP effect at a constant time, and the pressure effect on the samples was studied only after HPP treatment. To better understand the HPP effect on PBP extract stability and to better underpin the potential advantage of HPP over thermal pasteurization, as was qualitatively observed in Figure 3, we explored the in situ color deterioration kinetics under pressure and during heating, revealing for the first time the effect of HPP on PBP during the processing itself.

Due to a decrease in volume during HPP, an effective increase in concentration occurs, thus the absorption intensity might be slightly changed. In addition, a slight change in the optical pathway during pressure utilization may also occur [40]. Usually, the concentration of phycocyanin can be estimated with an equation using the ratio between the optical densities at 620 (the maximum absorbance of C-phycocyanin) and 652 (the maximum absorbance of allophycocyanin) [53]. However, due to the expected slight change in the absorption intensity and optical pathway, the equation cannot be used for C-phycocyanin degradation rate calculation at high pressure. Thus, we followed the color deterioration rate by following the absorption decrease at 620 nm. Similar to the reported phycocyanin first-order degradation kinetics at high temperatures [17,19] we observed that PCB extract empirically follows first-order kinetics for color deterioration at high pressure. However, the color deterioration mechanism might be substantially different between the treatments. Color deterioration rate (CDR) was calculated by the following equation:

(1) $$\left({\mathrm{At}}_{620\mathrm{nm}}\right)=\left(\mathrm{At}0_{620\mathrm{nm}}\right){*e}^{-\mathrm{CDR}*t}$$

where ${\mathrm{At}}_{620\mathrm{nm}}$ is the absorbance at 620 nm at time t, $\mathrm{At}0_{620\mathrm{nm}}$ is the absorbance at 620 nm at time t = 0 and CDR is the color deterioration rate.

**Figure 4:**
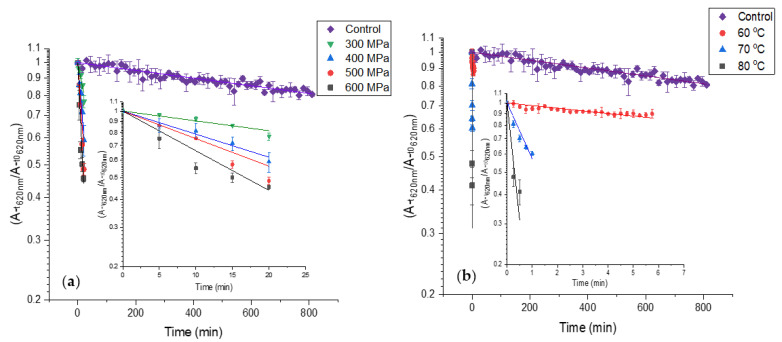
First-order color deterioration kinetics of PBP extract at high pressure and temperature (control treatment at atmospheric pressure 25 °C in baroresistant buffer pH 7 100 mM). The small figures present the results without PCB extract at 0.1 MPa 25 °C while the trendlines represent fit to first-order color deterioration kinetic) (**a**) high-pressure (**b**) high temperature. The temperature at high pressure was kept constant at 25 °C. (*n* = 2, error bars represent S.E.).

The HPP effect on PCB extract color deterioration is presented in Figure 4a and Table 1. The results reveal that the CDR of PCB extract increases substantially at high pressures. Among the pressures, the CDR increases moderately. The heat effect on PCB extract color deterioration is presented in Figure 4b and Table 1. The results reveal that heat treatment causes fast color deterioration of PCB extract and temperature increase causes a substantial CDR increase. The CDR at 70 and 80 °C is substantially higher than the CDR at HPP. Considering the color degradation rates and the standard processing times for pasteurization-level treatments (3–5 min at 500–600 MPa for HPP, 0.5–2 min 70 80 °C for thermal treatment), some advantages for HPP over thermal pasteurization in the color preservation of PBP extract containing products can be suggested. 

### 3.4. The Effect of pH on PCB Extract Stability 

**Figure 5:**
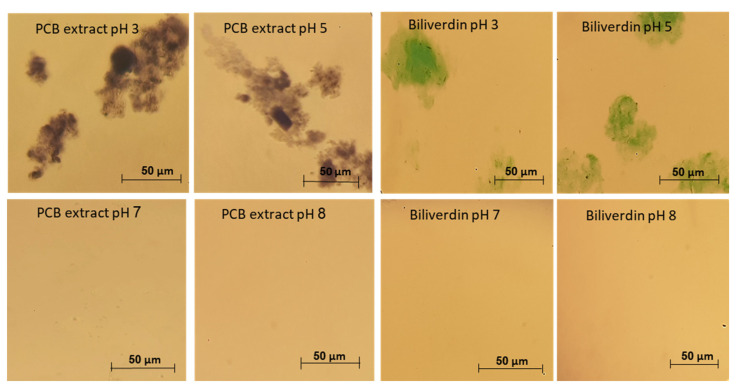
Typical light microscope pictures of biliverdin and PCB extract at different pH.

In Section 3.2 we have revealed that PCB extract and biliverdin have remarkable heat and pressure stability, thus from stability to processing point of view, extracted PCB can be a potential blue food pigment. On the other hand, to the best of our knowledge, the solubility and stability of phycocyanobilin and biliverdin in aqueous solutions have not been studied before. This knowledge is crucial to enable the use of PCB as a food pigment. We have studied the stability of PCB extract and biliverdin at different storage pH, in a concentration where the absorbance of the samples at 260–800 nm was below 1. However, aggregates were formed when PCB extract and biliverdin were added to the buffer at pH 3 and 5, resulting in difficulty in filtering the solutions through a 0.45 μm PVDF syringe filter prior to LC-MS analysis. Figure 5 presents light microscope images of PCB extract and biliverdin at various pH levels. The figure reveals that the pigments aggregate extensively at pH 3 and 5. In addition, Figure S3 (Supplementary Materials) presents the spectrum of PCB extract and biliverdin at different pH after centrifugation. The figure reveals significantly lower absorbance of PCB extract and biliverdin at pH 3 and 5, in addition to some spectral shifts in the UV and visible ranges, while at pH levels 7 and 8, the examined compounds present high solubility.

PCB has a predicted pK_a1_ of 2.4. After monoanion formation, an intramolecular hydrogen bond is formed between the ionized and the protonated group, keeping PCB in a tight conformation. This tight conformation prevents the second ionization of its carboxylic group. Thus, the estimated pK_a2_ of PCB is 9.6. In addition, this confirmation might also limit the PCB intermolecular bonds with water, potentially limiting the solubility in this conformation. In the dianion form, the internal hydrogen bond keeping PCB in its tight conformation is expected to disconnect [54]; intermolecular bonds between water and PCB are expected to be formed, and the PCB solubility is expected to increase. The predicted pKa is often higher than the measured one [55]. Therefore, although PCB has predicted pK_a2_ of 9.6, at pH 7–8 PCB might be in its dianion form. Biliverdin has a similar structure as PCB [54], and it is expected to have a similar conformation as PCB during ionization and similar interactions with water are expected.

**Figure 6:**
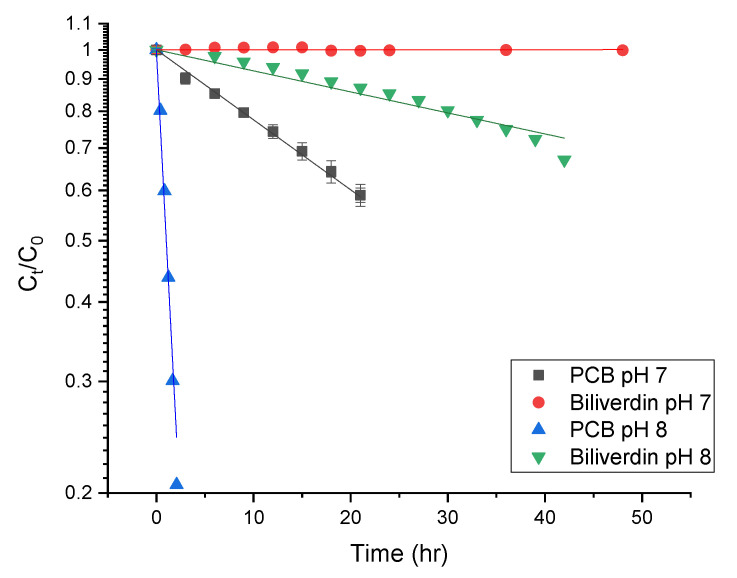
First-order degradation kinetics of biliverdin and PCB at pH 7 and 8 (*n* = 3, the lines represent fit to first-order degradation kinetic).

While the pH of 7–8 is slightly higher than in most foods [56], we focused on revealing the stability of the pigment at these pH levels due to the pigment’s increased solubility, hypothesizing that at slightly more acidic pH levels, common to food products, the degradation rate might change, however similar oxidation pathways may occur. Figure 6 presents the degradation rate of PCB and biliverdin at pH 7 and 8. The figure reveals that the pigments empirically follow a first-order degradation reaction. The first-order degradation rate was calculated using:

(2) $$\;\left(C_{t}\right)=\left(C_{0}\right){*e}^{-K*t}$$

where $C_{t}$ is the concentration at time t measured as the area under the curve at 270 nm, $C_{0}$ is the concentration at time t = 0 (measured with the same method as $C_{t})\;\mathrm{and},\;K$ is the degradation rate constant. Table 2 presents the degradation rate and half-life times of biliverdin and PCB at different pH. The table reveals that as the pH increases from 7 to 8, the pigments degradation rate also increases. PCB had a substantially higher degradation rate at both pH levels than biliverdin. The difference in the degradation rate of the pigments is a result of their structure. We suggest that it stems from the vinylic group on the γ-lactam ring in biliverdin, while PCB has an allylic group. Hydrogen abstraction from an allylic group by radicals is usually easy as the formed allylic radical is stabilized by resonance. Thus, it is expected that the allylic group would be easily oxidized. However, hydrogen abstraction of the vinylic group is more difficult as the formed vinylic radical is highly unstable [14,15]. Such results might suggest that contrary to the clear impact of protein denaturation on the PBP color loss during processing, the observed degradation of PCB might indicate that the reported color deterioration of PBP extract at room temperature, as also seen in Section 3.3 (25 °C and 0.1 MPa) is a result of pigment oxidation. Oxidation was reported previously as a possible reaction limiting the storage stability of phycocyanin [31].

**Figure 7:**
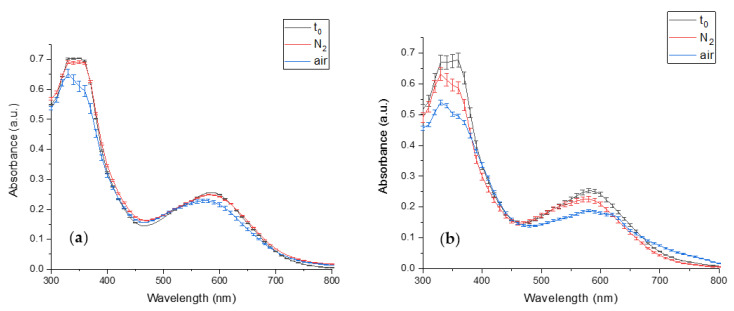
The effect of different storage environments on PCB extract degradation (t_0_- PCB extract absorbance in time 0 (min), N_2,_ and air, the absorbance after storage in an inert and atmospheric environment, respectively) (**a**) the absorbance at pH 7 after 29 (h) (**b**) the absorbance at pH 8 after 2 (h). (*n* = 2, error bars represent S.E.).

The PCB extract degradation in an inert and atmospheric environment was studied to verify that PCB degradation is a result of oxidation. Because of the high detected biliverdin stability (Figure 6), its oxidation was not studied. Figure 7a presents the effect of the different storage conditions on PCB extract degradation at pH 7. The figure reveals that while the exposure of PCB extract to air results in a significant absorbance decrease, the absorbance of PCB extract in an inert environment (N_2_) remains almost unaltered during the storage conditions (for 29 (h)). Thus, it may be concluded that the absorbance decrease seen in the atmospheric environment results from oxidation. At pH 8 the absorbance of PCB extract in an inert environment decreased during the storage (for 2 (h)) (Figure 7b), but still, PCB extract had a much more extensive decrease in the atmospheric environment due to oxidation. The decrease in PCB extract absorbance in the inert environment might result from other reactions, such as basic hydrolysis. 

## 4. Conclusions

The research has focused on the effect of different storage and pasteurization conditions on the stability of phycocyanobilin (PCB) extract and phycobiliproteins (PBP) extract. First, the effect of heat and high pressure on the stability of PBP extract, PCB extract, and biliverdin was studied. It was revealed that phycobiliproteins denaturation and color deterioration occurred during high-pressure and heat treatment. An increase in both pressure and temperature level resulted in an increased color deterioration rate in situ. The kinetics of color change during processing suggest that there are some advantages for HPP over thermal pasteurization in the color preservation of PBP extract-containing products, yet this should be verified for each specific product based on the formulation and required processing intensity. On the other hand, phycocyanobilin extract and biliverdin remained stable during processing, thus confirming that protein denaturation and not pigment oxidation is the main factor for the chemical instability during the processing of phycobiliproteins and suggesting that PCB is a potential food pigment from the stability point of view. Yet, during storage at pH 7, 8 rapid PCB oxidation occurred, while at lower pH levels, PCB had limited solubility, and aggregation of the pigment was detected. Therefore, in addition to evaluating the economic feasibility of its utilization, its solubility must be improved to implement PCB as a food pigment. 

## Figures and Tables

**Table 1:** Color deterioration rate at different pressures and temperatures ^1,2^.

**Pressure (MPa)**	**CDR (1/min) × 10^2^**
0.1	0.0024 ± 0.0008 ^a^
300	1.4 ± 0.1 ^b^
400	1.7 ± 0.5 ^b^
500	3.4 ± 0.1 ^b^
600	4.7 ± 0.1 ^c^
**Temperature (°C)**	**CDR (1/min) × 10^2^**
25	0.0024 ± 0.0008 ^a^
60	2.0 ± 0.1 ^b^
70	58 ± 3 ^c^
80	227 ± 7 ^d^

^1^ Different letters indicate significant (*p* < 0.05) differences between rows based on the nonlinear fit, at least 5 data points per fit. ^2^ The statistical significance was calculated separately for high pressure and high temperature due to the possible different deterioration mechanisms.

**Table 2:** Degradation rate and half-life time of biliverdin and PCB extract at pH 7 and 8 ^1^.

Solution	Degradation Rate (1/h) × 10^2^	Half-Life Time (h) × 10^3^
PCB extract pH 7	2.5 ± 0.2 ^a^	2.7 ± 0.2 ^a^
PCB extract pH 8	9.4 ± 0.006 ^b^	0.74 ± 0.01 ^b^
Biliverdin pH 7	ND ^2^	ND ^2^
Biliverdin pH 8	0.76 ± 0.01 ^c^	9.1 ± 0.1 ^c^

^1^ Different letters indicate significant (*p* < 0.05) differences between rows based on the nonlinear fit, at least 6 data points per fit. ^2^ The degradation rate of biliverdin at pH 7 was not detected during the experiment.

## Data Availability

Data is contained within the article or Supplementary Materials.

## Supplementary Materials

The following are available online at https://www.mdpi.com/article/10.3390/antiox12030568/s1, Figure S1: The effect of HPP at different pressures on PBP extract, PCB extract, and biliverdin. Figure S2: The effect of different high-temperature pasteurization conditions on PBP extract, PCB extract, and biliverdin. Figure S3: The spectrum of PCB and biliverdin after centrifugation at different pH levels.
