# Supplementary material for: The Effect of Storage and Pasteurization (Thermal and High-Pressure) Conditions on the Stability of Phycocyanobilin and Phycobiliproteins
Source: Antioxidants (Basel). 2023 Feb 24;12(3):568. doi: 10.3390/antiox12030568 (PMC10045346; doi:10.3390/antiox12030568)
Supplement: Supplementary file 1 [file antioxidants-12-00568-s001.zip › antioxidants-2183726-supplementary.pdf]

antioxidants

MDPI

Supplementary Materials

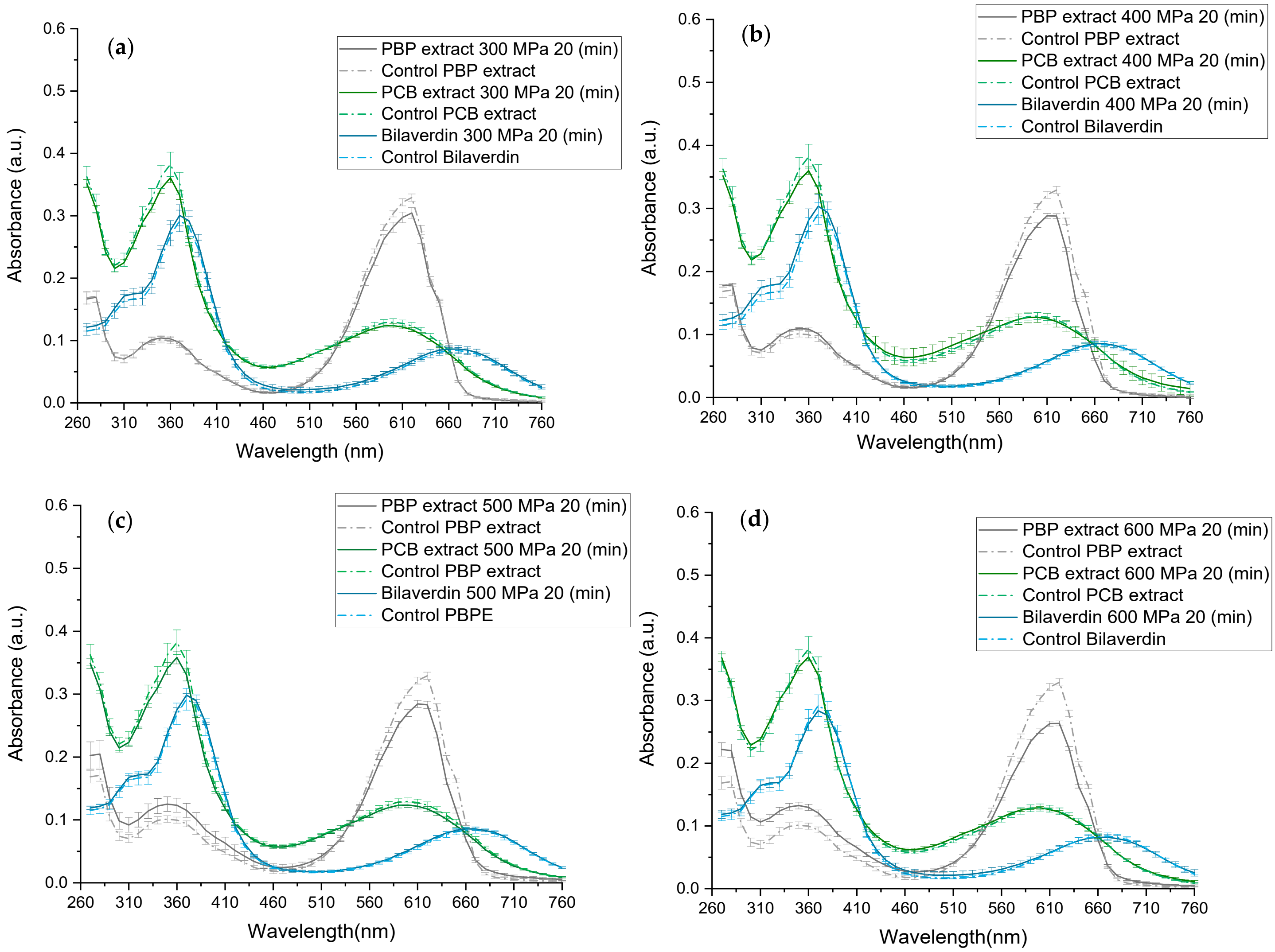

Figure S1. The effect of HPP at different pressures on PBP extract, PCB extract and biliverdin (control- treatment at atmospheric pressure 25 °C)(**a**) 300 MPa (**b**) 400 MPa (**c**) 500 MPa (**d**) 600 MPa. (n=3, errors represent S.E)

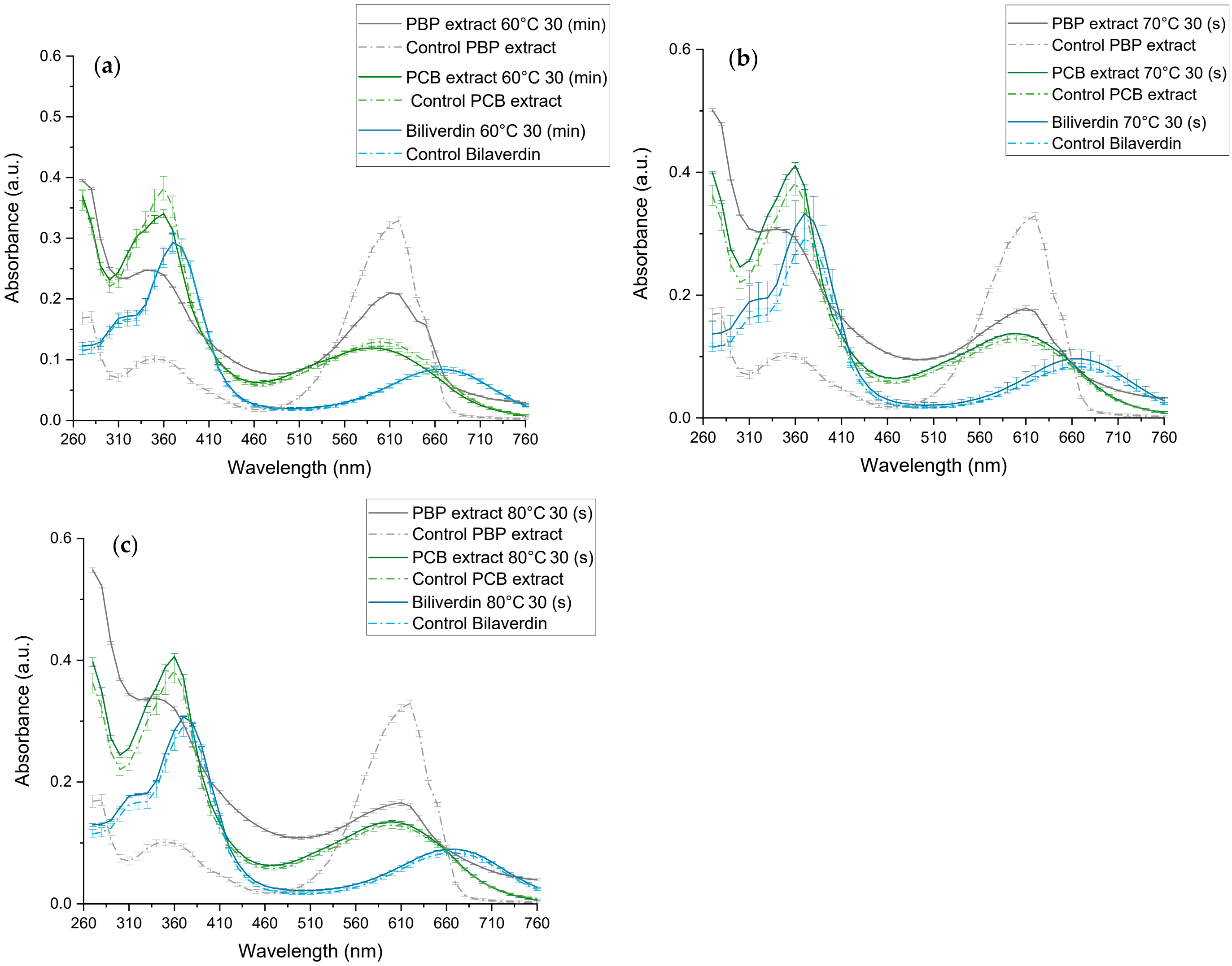

Figure S2. The effect of different high-temperature pasteurization conditions on PBP extract, PCB extract and biliverdin (control-treatment at 25 °C)pH 7 (**a**) 60 °C (**b**) 70 °C (**c**) 80 °C. (n=3, errors represent S.E)

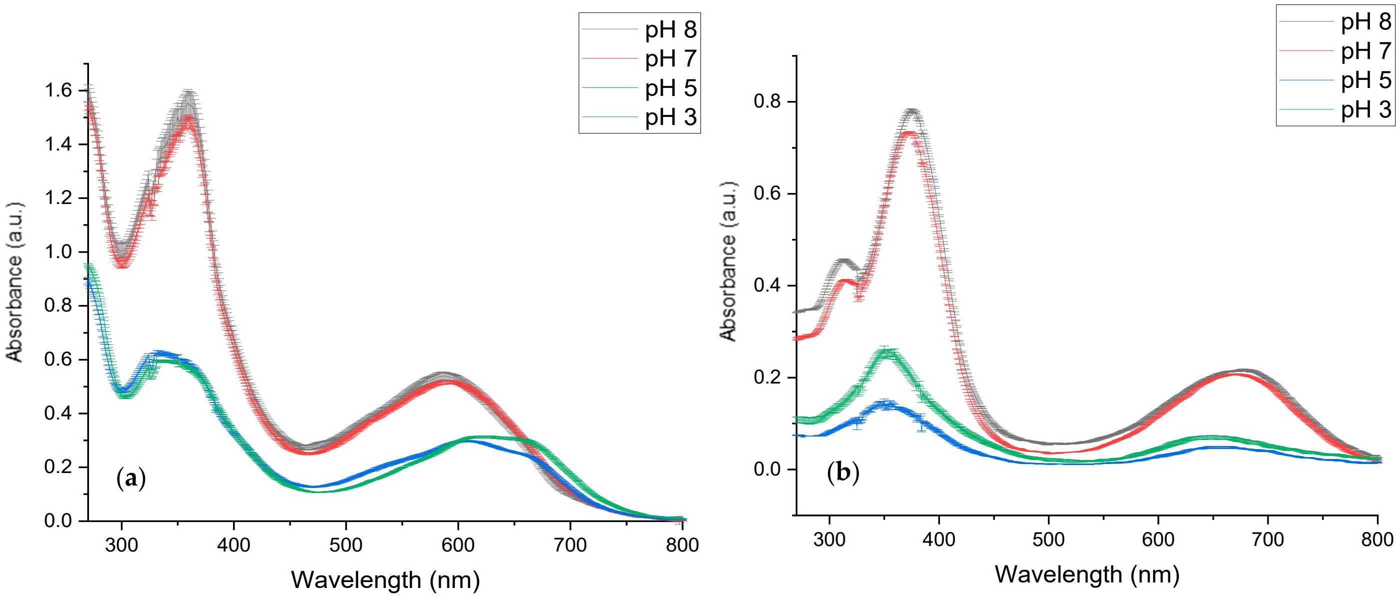

Figure S3. The spectrum of PCB and biliverdin after centrifugation at different pH levels (**a**) PCB extract (**b**) biliverdin. (n=3, errors represent S.E)
